# The National Medical Union's Latest Undertaking

**Published:** 1914-01-31

**Authors:** Robert Carswell, E. Arthur Dorrell

**Affiliations:** Hon. Secretary; Hon. Secretary


### THE PROFESSION OF MEDICINE.

### The National Medical Union's Latest Undertaking.

The work of the National Medical Union has increased to a stage at which it has become necessary to economise by division of labour. One of the chief duties entrusted to the Council by the Conference was the enrolment of members of the medical profession not on the panel and in sympathy with the objects and policy of the Union, and the Executive Committee now considers it expedient to recommend the formation of a Special Organisation Committee for this purpose alone. In certain districts of London and elsewhere the local Associations have actively carried on this work in their own neighbourhood. They have issued local prospectuses, obtained complete lists of the non-panel doctors of the district, and with the aid of these have instituted a systematic canvas, dividing up the work amongst their local Committees.

It is only by personal interviews that the interest of those who are not in touch with what is being done can be aroused. Amongst those who are fully aware of the trend of events and of the ideas which are animating the Union there is no lack of enthusiasm, but the medical profession generally has become apathetic on the subject of combination and nothing short of personal influence is sufficient to arouse interest. The formation of a Central Organisation Committee to take charge of this department will enable the whole field to be dealt with without the interruption due to the introduction of other issues which the Executive Committee, as such, has unavoidably to consider. Too much importance cannot be attached to the efficient discharge of this duty. At the present time there are a number of indications that changes in the panel system are not far off, and the Union may be confronted with a situation which will call for action sooner than has been anticipated.

Another duty of the Council which calls for special treatment, and which is of equal or even greater importance, is that of drafting proposals for a constitution. This will demand much careful consideration, and will probably be most efficiently performed by a small Committee, meeting frequently and collecting reference material from all sources. The difficulties which have beset the British Medical Association in connection with its constitution are well known, and the Union may well profit by its experience. In addition to the model of the British Medical Association there are other types of constitution to be taken into account. The question will arise whether an organisation such as the National Medical Union ought not to be armed with every possible weapon suitable for a body which will undoubtedly have to adopt a fighting policy.

The attention of members of the Union is specially directed in this connection to a book entitled "Medical Benefit: a Study of the Experience of Germany and Denmark," by I. G. Gibbon.* Compulsory insurance against sickness has been in existence in Germany since 1884, and a voluntary scheme has existed in Denmark since 1893. These schemes are compared and contrasted, and certain conclusions are drawn as to the most efficient method of dealing with the subject which should prove of the deepest interest and value to members of the Union. In the appendices much valuable information on the subject of the organisation of medical practitioners will be found. It is stated that the Leipzig Union is frankly a trade union, and that it contains 95 per cent. of the medical practitioners in Germany. The origin of this Union in 1900 corresponds very closely with that of our National Medical Union. A Society similar to the British Medical Association had been in existence in Germany since 1873, but in consequence of the pressure of the German insurance laws the Leipzig Union took a separate existence. It is noteworthy that three years after its formation it joined hands with the earlier Association in an arrangement which relegated to the Leipzig Union the protection of economic interests, while the older Association remained purely a scientific body.

* P. S. King and Son, Orchard House, Westminster, price 6s.

### The Manchester Meeting.

Dr. Prowse, of Manchester, writes: "The Executive Committee of the National Medical Union (Manchester District) held a meeting on January 12. There was an interesting discussion on policy, which resulted in the passing of a resolution calling attention to the urgent need for the adoption of a definite policy, and suggesting that the Council should take steps to promote the amendment of the existing Insurance Acts, or the drafting of a new Bill more in consonance with the views of the medical profession.

"The Joint Committee of the Manchester and Salford Divisions of the British Medical Association arranged a meeting of the profession for Tuesday, January 13, to hear an address on 'Trade Unionism for the Medical Profession' from Dr. Percy Raiment, Hon. Secretary of the National Medical Guild. The meeting took place in the large Memorial Hall under the chairmanship of Dr. Arthur Helme. Between thirty and forty men were present. One of the aims of the Guild is to obtain recognition and support from the British Medical Association—with the view, apparently, of taking over the medico-political work of that body.

"The National Medical Union of Manchester, since its inception two years ago, has been opposed to the idea of trade unionism for medical men. Coercive methods founded chiefly on financial and other considerations, which do not appeal to the highest instincts of men, are not likely to form a bond of union which will be an effectual weapon in fighting for the true interests of the profession."

Robert Carswell, } Hon. Secretaries.

E. Arthur Dorrell, }

